# Critical appraisal of clinical practice guidelines for treatment of urinary incontinence: Protocol for a systematic review

**DOI:** 10.1097/MD.0000000000016698

**Published:** 2019-08-16

**Authors:** Flavia Blaseck Smiles, Lauren Giustti Mazzei, Luciane Cruz Lopes, Silvio Barberato-Filho, Juliana Castro, Analaura Castro, Livia Luize Marengo, Cristiane Cássia Bergamaschi

**Affiliations:** Graduate Program in Pharmaceutical Sciences, University of Sorocaba, Sorocaba, São Paulo, Brazil.

**Keywords:** health planning guidelines, systematic review, urinary incontinence

## Abstract

**Background:**

Urinary incontinence is a common complaint in all parts of the world, cause of distress, as well as significant costs for both individuals and society. The aim of this study will be to evaluate the rigor of the development of clinical practice guidelines and to identify the recommendations of interventions for urinary incontinence in adult women.

**Methods:**

In this systematic review, clinical practice guidelines will be identified using a prospective protocol through a systematic search of: MEDLINE (via Ovid); EMBASE (Excerpt Medical Database, via Ovid); Web of Science and Virtual Health Library. Specific databases of guidelines for clinical practice will also be searched (National Institute for Health and Care Excellence, American Urological Association, and others). Reviewers, independently and in duplicate, will assess the quality of the guidelines using the Appraisal of Guidelines Research and Evaluation (AGREE II). The results will be checked for discrepancies. Differences between the scores equal to or greater than 2 will be considered as discrepant and the final result will be decided by consensus. A comparison of the recommendations of interventions and information about the level of evidence, the degree of recommendation, the level of agreement and the level of acceptance will be described. This step will also be done independently and in duplicate, and the result will be decided by consensus. The results will be presented in tables and the descriptive statistics will be calculated for all domains of the AGREE II instrument as mean (standard deviation) and median (interquartile range).

**Results:**

The results derived from this study will increase the knowledge about the development of recommendations guidelines for urinary incontinence of high methodological rigor. This study may also identify key areas for future research.

**Conclusion:**

This study may guide health professionals, policy makers, and health policy managers in choosing the guidelines for recommendation in clinical practice.

**Protocol Registration:**

PROSPERO - CRD42018116517

## 1 Introduction

Urinary incontinence is a common complaint in all parts of the world, a cause of distress, as well as significant costs for individuals and for society.^[1]^ It has been associated with significant physical morbidity, loss of independence, decreased quality of life, and participation in social and domestic activities.^[2]^

The classification of urinary incontinence varies according to the patient's symptoms. Urge incontinence is present when there is a report of involuntary leakage associated or immediately preceded by a sudden need for emptying without the ability to delay.^[3]^ The involuntary effort leakage complaint performed on some type of activity is considered as stress urinary incontinence. When there is involuntary leakage associated with urgency and effort, urinary incontinence is classified as mixed.^[4]^

The prevalence among types of urinary incontinence varies among countries. It is estimated that women are more affected than men, and between 10% and 55% with ages between 15 and 64 years are affected, with the highest prevalence of stress urinary incontinence.^[4]^ The number of women with urinary incontinence tends to increase along life expectancy, especially in middle age, when cases become more prevalent.^[5]^

The history of urinary incontinence is fundamental to the planning of the clinical process and should be the first step in the evaluation, informing details about the type, moment, severity, and other symptoms, allowing the categorization of the disease.^[6]^ Although history provides pertinent data on urinary incontinence, it is often the case that the diagnosis is not complete, since urinary symptoms may be similar, so physical examination is required as part of urogynecologic evaluation of the patient.^[5]^

Treatment options for urinary incontinence may be surgical or conservative. In clinical practice, non-surgical therapies are the first line of treatment, including behavioral therapy with strategies for re-education of the bladder, training of pelvic muscle tone, biofeedback, electrical stimulation, vaginal cones, control of intake of caffeine, and pharmacological treatment, varying according to each case or type of urinary incontinence.^[5,7,8]^ Due to numerous options of treatments, many professional organizations have developed guidelines to help clinicians treat patients with urinary incontinence.^[7]^

Guidelines are important vehicles of influence for clinical practice. Local, national, and international societies adopt the process of identifying relevant clinical areas, formulating specific clinical issues, reviewing applicable evidence, and formulating recommendations that doctors and their patients should follow.^[9]^

To ensure reliability, clinical practice guidelines should be systematically developed by groups of people with skills, perspectives and knowledge based on the best available evidence.^[10]^ With the elaboration of these documents, the concerns related to their quality increased.^[10,11,12]^

The Appraisal of Guidelines for Research & Evaluation (AGREE II) Instrument aims to address the variability in the quality of clinical practice guidelines, that is, assesses the methodological rigor and transparency with which the guideline is developed. Developed by an international group, first published in 2003 and updated in 2009, AGREE II has been widely used, offering a comprehensive, rapid, and consistent assessment of clinical practice guidelines.^[13]^

No systematic review performed the critical appraisal on the development of clinical practice guidelines for the treatment of urinary incontinence. Success in implementing recommendations should be related to the use of appropriate methodologies and rigorous strategies in the guideline development process.^[13]^ The present study will evaluate the rigor of the development of clinical practice guidelines and will identify, in these documents, the recommendations of interventions for urinary incontinence in adults.

## 2 Methods

### 2.1 Study design

This systematic review of clinical practice guidelines for adult urinary incontinence interventions will be undertaken to assess the methodological quality in their development and the recommendations of the interventions, available in those documents.

### 2.2 Protocol and registration

This study will be reported according to the Preferred Reporting Items for Systematic Reviews and Meta-Analyses (PRISMA-P).^[14]^ The systematic review was register in the International Prospective Register of Systematic Reviews (PROSPERO) database (protocol number: PROSPERO - CRD42018116517), available in (https://www.crd.york.ac.uk/prospero/display_record.php?RecordID=116517). Ethical approval is not required because this is a literature-based study.

### 2.3 Eligibility criteria

#### 2.3.1 Inclusion criteria

Guidelines for clinical practice and consensus (if applicable) describing interventions for treatment of adults (age ≥ 18 years) with urgency, stress, or mixed urinary incontinence, even if the document reports 1 or more types of incontinence, will be included. We will consider the documents published from 2009 onwards (date of publication of the latest version of AGREE II), and restricted to English, Portuguese, Spanish, and French.

#### 2.3.2 Exclusion criteria

Specific clinical practice guidelines for the treatment of urinary incontinence due to neurological or oncological traumas will be excluded. If there is another more up-to-date version of the guideline; the available version is incomplete or contains only a summary of the information; the document is the translation of a guideline published in another language; and if there is a consensus guideline, evidence summary or algorithm; will be excluded.

### 2.4 Measured outcomes

The methodological quality of clinical practice guidelines for interventions for urinary incontinence in adults will be evaluated; the scores of each domain associated with the methodological quality of the guidelines will be identified; and the recommendations provided by the guidelines will be described and compared.

### 2.5 Selection of studies

#### 2.5.1 Search methods

The following electronic databases will be searched: MEDLINE (via Ovid); EMBASE (Excerpta Medical Database, via Ovid); Web of Science; and Virtual Health Library.

Specific databases for clinical guidelines will be searched, for example: ECRI Institute (www.guidelines.ecri.org) European Association of Urology (www.uroweb.org), NICE (www.nice.org.uk), American College of Physicians (www.acponline.org), American Urological Association (www.auanet.org), Brazilian Society of Urology (www.sbu-sp.org.br), Canadian Agency for Drugs and Technologies in Health (www.cadth.ca), Canadian Medical Association (www.cma.ca), and others.

#### 2.5.2 Other search features

The reference list of eligible studies, review studies, and secondary studies will be checked by reviewers in order to identify other possible guidelines. For guidelines published only in summary or where important information is missing, we will try to search complete information by contacting the authors.

#### 2.5.3 Search strategies

The key words will be used according to the terms of the Medical Subject Headings (MeSH) to identify relevant studies. The isolated terms and their entry terms will be identified and will be crossed to perform the search, being adapted for each database. The terms used will be: (Guideline OR Guidelines OR Practice Guideline OR Health Planning Guidelines OR Health Planning Guidelines OR clinical practice guidelines OR best practice OR best practices) AND (Urinary Incontinence OR Incontinence, Urinary OR Urinary Incontinence Urge OR Urinary Reflex Incontinence OR Incontinence, Urinary Reflex OR Urinary Urge Incontinence Urinary Urge Incontinence OR Urge Incontinence OR Incontinence, Urge OR Urinary Incontinence, Stress OR Urinary Stress Incontinence OR Incontinence, Urinary Stress OR Stress Incontinence, Urinary OR Lower Urinary Tract Symptoms OR Female Urogenital Diseases OR Urologic Diseases OR Urination Disorders OR Urological Manifestations). The search strategy will be adapted to each database.

### 2.6 Determination of eligibility

Duplicates will be removed by 1 of the reviewers. Reviewers (LLM and APMVC, LGM and JPMVC, FBS, and SB-F), in pairs and independently, will assess whether abstracts and titles meet the eligibility criteria.

The eligibility of the guidelines will be confirmed after reading the full text by the same reviewers and independently. Discrepancies will be solved by consensus and a third reviewer (CCB or LCL) will be able to assist in the final decision if necessary. In case of duplicate publication, the most up-to-date guideline will be used. All documents related to the guidelines (cited as supplemental documents, summaries of recommendations, and others) will be searched manually by 1 or 2 reviewers.

### 2.7 Data extraction

The information will be added to an Excel worksheet and the same reviewers, in pairs and independently, will be the extraction of the date. The discrepancies will be resolved by consensus. If cannot be resolved through discussion, will be referred to a third reviewer (CCB or LCL). Previously, the reviewers will be calibrated by extracting at least 3 documents of different levels of quality and will reach consensus. The results will be discussed with another reviewer, previously trained. This procedure should occur until the reviewers are able to extract the data.

The following data will be extracted: number of authors, year of publication, update time, organizations (government, medical society, university or other), type of guideline (formulated, adapted, updated or revised), country of development, type (diagnosis, prevention, pharmacological and non-pharmacological treatment, and/or other), type of urinary incontinence, treatments described, target population, design of studies included (systematic review, consensus, overview of systematic reviews, and/or other), methods of recommendation formulation (consensus, not mentioned, others), and methods of classifying the quality of evidence (GRADE, Oxford, not mentioned, or other).

### 2.8 Quality assessment of clinical practice guidelines

The quality of each guideline will be evaluated using the Appraisal of Guidelines for Research and Evaluation - AGREE II. The translated and validated version of AGREE II for the Portuguese language (Brazil) will be used. The tool consists of 23 items covering 6 quality domains, scored with a Likert scale of 1 (totally disagree) to 7 (totally agree) for each (Khan and Stein, 2014). The 6 areas are:

1. scope and purpose
2. stakeholder involvement
3. rigour of development
4. clarity of presentation
5. applicability
6. editorial independence.^[13]^

The same pairs of reviewers will conduct the quality assessment of the guidelines and the difference of 2 or more scores for each item will be considered as discrepant. The final score will be decided by consensus and if there is no consensus, another reviewer will help in the final decision.

The quality of each guideline will be calculated for each domain, according to the AGREE II User Manual. The 6 domains are independents and the scores should therefore be calculated as the sum of the individual items in each domain. Then, the total obtained will be presented as a relation percentage to the maximum possible score for each domain. The evaluation will be conducted using the “My AGREE PLUS” platform.^[13]^

Previously, a training will be done to use the AGREE II instrument according to the following steps:

1. study the AGREE II User Manual, the AGREE II validation article in Brazil and a guideline to choose;
2. register on the “My AGREE PLUS” platform and complete the AGREE II Training Tools (https://www.agreetrust.org);
3. calibration of the reviewers as previously described.

### 2.9 Description and comparison of the recommendations of the interventions

The study will describe and compare the recommendations of intervention: pharmacological, conservative (such as behavioral therapy with strategies for re-education of the bladder, training of pelvic muscle tone, biofeedback, electrical stimulation, vaginal cones, and others), and/or surgical using selected guidelines, respecting the particularities of the treatment of these diseases.

For the recommendations of the description and comparison of the intervention, the level of evidence supporting them will be found. The information will be collected in relation to this level of evidence, the degree of recommendation, the level of agreement and the level of acceptance.

This step will also be done in duplicate and independently by all reviewers. The information will be verified and, if there is no consensus, another reviewer will assist in the final decision.

### 2.10 Data synthesis

The results will be presented in descriptive tables. Descriptive statistics will be calculated for all AGREE II domains as mean (standard deviation) and median (interquartile range). Graphs will be plotted when needed. The level of significance will be 5%. Statistical analysis will be conducted using the STATA software (version 14.2).

## 3 Discussion

This study will identify guidelines of high-quality clinical practice describing interventions for urinary incontinence or the possible flaws observed in these articles. The results observed may guide the development of recommendations guidelines for urinary incontinence of high methodological rigor. Success in implementing recommendations should be related to the use of appropriate methodologies and rigorous strategies in the guideline development process.

A description of the available recommendations on interventions and evidence supporting them contributes to the choice of treatment for urinary incontinence in adults. Thus, the results of this study can subsidize patients, health institutions, health policy makers, choose higher quality guidelines, inform on the existing recommendations of the different interventions and identify gaps in current evidence and make recommendations for future research.

The method of this review includes explicit eligibility criteria, comprehensive and extensive database research, independent, and paired evaluation for study selection. Nevertheless, the fact of the present study will be limited to subjective analysis of the AGREE II instrument may be a limiting factor.

The results of the research can be submitted for publication in scientific journals of high impact, peer reviewed, and also published in national and international conferences.

## Author contributions

**Conceptualization:** Flavia Blaseck Smiles, Cristiane Cássia Bergamaschi.

**Investigation:** Luciane Cruz Lopes, Silvio Barberato-Filho.

**Methodology:** Flavia Blaseck Smiles, Lauren Giustti Mazzei, Luciane Cruz Lopes, Silvio Barberato-Filho, Juliana Castro, Analaura Castro, Livia Luize Marengo, Cristiane Cássia Bergamaschi.

**Supervision:** Cristiane Cássia Bergamaschi.

**Writing – original draft:** Flavia Blaseck Smiles, Lauren Giustti Mazzei, Luciane Cruz Lopes, Silvio Barberato-Filho, Juliana Castro, Analaura Castro, Livia Luize Marengo, Cristiane Cássia Bergamaschi.

**Writing – review & editing:** Lauren Giustti Mazzei, Luciane Cruz Lopes, Silvio Barberato-Filho, Juliana Castro, Analaura Castro, Livia Luize Marengo, Cristiane Cássia Bergamaschi.

Cristiane Cássia Bergamaschi orcid: 0000-0002-6608-1806.
