# Critical appraisal of clinical practice guidelines for treatment of urinary incontinence: Protocol for a systematic review: Erratum

**DOI:** 10.1097/MD.0000000000017085

**Published:** 2019-08-30

In the article, “Critical appraisal of clinical practice guidelines for treatment of urinary incontinence: Protocol for a systematic review”,^[1]^ which appears in Volume 98, Issue 33 of *Medicine*, Dr. Flavia Blaseck Sorrilha's name appears incorrectly as Flavia Blaseck Smiles.
